# Choline Lactate Photocured Hydrogels for Sustainable Low-Temperature Supercapacitors

**DOI:** 10.3390/gels12070623

**Published:** 2026-07-10

**Authors:** Joanna Fijałkowska, Julianna Czerniawska, Beata Sikora, Wiktoria Patz, Julia Marecka, Łukasz Popenda, Piotr Gajewski, Katarzyna Szcześniak, Agnieszka Marcinkowska

**Affiliations:** 1 Institute of Chemical Technology and Engineering, Poznan University of Technology, Berdychowo 4, 60-965 Poznan, Poland; joanna.fijalkowska@student.put.poznan.pl (J.F.); julianna.czerniawska@student.put.poznan.pl (J.C.); beata.sikora@student.put.poznan.pl (B.S.); wiktoria.patz@doctorate.put.poznan.pl (W.P.); julia.marecka@student.put.poznan.pl (J.M.); piotr.gajewski@put.poznan.pl (P.G.); 2 NanoBioMedical Centre, Adam Mickiewicz University, Wszechnicy Piastowskiej 3, 61-614 Poznan, Poland

**Keywords:** hydrogel, hydrogel polymer electrolyte, supercapacitor, photopolymerization, choline lactate, green electrolyte

## Abstract

The growing demand for flexible and environmentally friendly energy storage systems has increased interest in new electrolyte materials capable of operating at low temperatures. In this work, hydrogel polymer electrolytes based on aqueous choline lactate solutions were developed and evaluated for supercapacitor applications. Choline lactate was synthesized from biodegradable and low-toxicity substrates and characterized using spectroscopic and thermal analysis methods. A series of aqueous electrolytes with different salt concentrations was prepared, and their viscosity, density, and ionic conductivity were investigated to determine the optimal composition for hydrogel preparation. The obtained hydrogels were synthesized by photopolymerization and showed good flexibility, transparency, and structural stability without electrolyte leakage. Thermal analysis revealed that the presence of choline lactate effectively suppressed water crystallization, reducing the phase transition temperature of the hydrogel systems below −44 °C. Ionic conductivity increased with electrolyte content and reached 22.3 mS·cm^−1^ at room temperature for the hydrogel containing 90 wt% electrolyte. Mechanical measurements showed that increasing electrolyte concentration improved flexibility but reduced stiffness and compressive strength. Electrochemical tests demonstrated stable supercapacitor operation in the temperature range from 25 °C to −20 °C, although lower temperatures led to decreased capacitance and increased internal resistance. The results indicate that choline lactate-based hydrogels are promising candidates for sustainable low-temperature energy storage devices.

## 1. Introduction

Energy storage technologies are essential for the efficiency, reliability, and sustainability of modern energy systems [1]. The depletion of fossil fuel resources and the rapid development of wearable electronics, portable sensors, and flexible devices have accelerated the demand for environmentally friendly and mechanically durable energy storage systems [2,3,4,5]. In particular, flexible energy storage devices require stable and safe components, such as electrolytes and separators, capable of maintaining performance under mechanical deformation [6].

Among conventional energy storage technologies, supercapacitors (SCs) have attracted considerable attention because of their high power density, rapid charge–discharge capability, and excellent cycling stability exceeding 100,000 cycles [7]. Their ability to rapidly deliver electrical energy makes them attractive for portable electronics, wearable devices, and other applications requiring fast power delivery [1,8,9]. Electrochemical double-layer capacitors (EDLCs), which store energy through electrostatic ion adsorption at the electrode–electrolyte interface, are particularly promising due to their simple and safe configuration compared with lithium-ion batteries [3,10,11].

The electrolyte and separator are key components determining SC performance. Separators must electrically isolate electrodes while allowing efficient ion transport, requiring high ionic permeability, chemical stability, and mechanical robustness [12]. Electrolytes strongly influence capacitance, power density, and cycle life through their ionic conductivity, viscosity, and thermal behavior [9,12,13,14,15]. To improve flexibility and safety, solid-state and gel polymer electrolytes (GPEs) have emerged as alternatives to liquid electrolytes [16]. However, their ionic conductivity often decreases significantly at low temperatures due to restricted polymer chain mobility, limiting supercapacitor performance in cold environments [1].

Low-temperature operation remains one of the major challenges limiting the practical application of supercapacitors [17]. The commercially available devices are typically limited to operating temperatures of −40 °C or higher and show substantial losses in capacitance and power below 0 °C [18,19,20]. Such limitations are particularly critical for applications in harsh cold environments where energy storage systems must rapidly deliver electrical energy under ultralow-temperature conditions [17].

The deterioration of electrochemical performance at low temperatures is mainly associated with increased electrolyte viscosity, reduced ionic conductivity, and restricted ion transport, which collectively decrease power density and overall device efficiency. Therefore, current research efforts are focused on extending the operational temperature range of supercapacitors while simultaneously improving their energy density, power density, reliability, and long-term stability [18,19,20]. In this context, environmentally friendly aqueous electrolytes based on cholinium salts have emerged as promising low-temperature systems. For example, a 5 mol·kg^−1^ aqueous cholinium chloride electrolyte demonstrated high conductivity of 88 mS·cm^−1^ at 24 °C and enabled carbon/carbon supercapacitor operation down to −40 °C while maintaining near-neutral pH conditions [18].

In recent years, choline-based ionic liquids (ILs) and deep eutectic solvents (DESs) have attracted increasing attention as environmentally friendly electrolytes for electrochemical energy storage devices. Choline-based compounds are considered particularly promising due to their low toxicity, biodegradability, cost-effectiveness, and simple synthesis routes compared with conventional imidazolium-based ionic liquids [21,22,23,24,25]. Choline chloride (ChCl), a quaternary ammonium salt belonging to the vitamin B family, is widely used in animal feed, human nutrition, pharmaceuticals, and fertilizer production, and is regarded as non-toxic and readily biodegradable [24,26]. Moreover, cholinium-based ionic liquids containing biodegradable organic anions, such as amino acid carboxylates or organic carboxylates, exhibit high biodegradability according to OECD standards [21].

Among choline-based electrolytes, choline lactate ([Chol][LA]) has emerged as a particularly attractive candidate for flexible and sustainable supercapacitors. Choline lactate combines the favorable electrochemical properties of ionic liquids with the biodegradability and biofriendliness of both choline and lactate ions [21,22]. Eco-friendly ionogels based on 2-hydroxyethyl cellulose (HEC) and biodegradable choline lactate have already been successfully applied in flexible and ultra-thin micro-supercapacitors operating above 1.5 V, demonstrating an electrochemical stability window of approximately 1.6 V [27,28]. The biodegradation behavior of both cellulose and choline lactate has also been extensively investigated and confirmed [21,22]. Furthermore, choline lactate and related choline-based systems can be synthesized through simple and low-waste acid–base reactions performed in aqueous media, supporting sustainable fabrication approaches [28].

Choline-based electrolytes have also shown promising electrochemical performance under challenging operating conditions. Choline chloride-based DES electrolytes have demonstrated excellent capacitance retention and cycling stability in supercapacitors [29], while ternary DES systems based on choline chloride enabled supercapacitor operation over a wide temperature range from −40 to 115 °C [30]. Similarly, aqueous choline nitrate-based electrolytes allowed stable carbon/carbon supercapacitor operation between −40 °C and 60 °C [31]. Owing to their low toxicity, biodegradability, favorable ionic conductivity, and potential for low-temperature operation, choline-based ionic liquids and DESs are increasingly considered promising electrolyte candidates for next-generation flexible and sustainable energy storage devices [23,29,30,31].

Previous studies have demonstrated the potential of choline-based ionic liquids and deep eutectic solvents as environmentally friendly electrolytes for electrochemical energy-storage devices. Choline lactate-based ionogels have been successfully applied in flexible micro-supercapacitors, while choline chloride- and choline nitrate-based electrolytes have enabled supercapacitor operation over extended temperature ranges, including subzero conditions [18,27,28,29,30,31]. These studies confirmed the advantages of choline-based systems in terms of ionic conductivity, electrochemical stability, biodegradability, and environmental compatibility. Furthermore, several studies have focused on the covalent incorporation of choline-based ionic species into hydrogel networks, leading to the formation of poly(ionic liquid) (PIL) hydrogels [32,33,34,35]. Representative examples include choline–amino acid poly(ionic liquids) [32] and methacrylated alginate functionalized with choline salts such as choline chloride, acetate, propionate, glycolate, succinate, and benzoate [33]. These materials generally exhibited satisfactory mechanical stability; however, they were often characterized by relatively high stiffness or limited elasticity and ionic conductivities below 1 S·m^−1^, with many systems showing values below 1 mS·m^−1^. Collectively, these studies demonstrate that choline-based ionic moieties can enhance the functionality of hydrogels, although their covalent immobilization may limit ion mobility and, consequently, the achievable ionic conductivity.

Despite the growing interest in choline-based ionic liquids and deep eutectic solvents for electrochemical energy storage systems, studies on choline lactate-based hydrogel separators remain limited. In particular, there is still insufficient understanding of the influence of choline lactate on low-temperature ionic conductivity, electrochemical stability, and mechanical durability of hydrogel systems under freezing conditions. These challenges are especially important for flexible supercapacitors intended for operation in harsh cold environments, where conventional aqueous electrolytes suffer from reduced ion mobility and loss of electrochemical performance. Therefore, the development of sustainable and anti-freezing hydrogel separators with stable conductivity, mechanical flexibility, and reliable electrochemical behavior at low temperatures remains highly desirable for next-generation supercapacitors.

In this work, hydrogel polymer electrolytes containing aqueous choline lactate electrolyte were developed and investigated for low-temperature supercapacitor applications. The study focuses on evaluating the influence of choline lactate on the physicochemical, mechanical, and electrochemical properties of the hydrogels, with particular emphasis on ionic conductivity, anti-freezing behavior, and performance stability under subzero operating conditions.

## 2. Results and Discussion

### 2.1. Electrolyte Preparation

#### 2.1.1. Choline Lactate Synthesis

The aim of this study was to obtain an environmentally friendly salt for the preparation of a green electrolyte, followed by the in situ synthesis of a hydrogel separator containing an aqueous solution of the synthesized salt via photopolymerization. Choline and lactic acid were selected as substrates for salt synthesis. As mentioned previously, choline is a naturally occurring, biodegradable, and biocompatible compound widely used in pharmaceutical applications and green chemistry. Similarly, lactic acid is a fully biodegradable and environmentally friendly substance commonly applied in the cosmetics, pharmaceutical, food, and agricultural industries.

**Figure 1:**
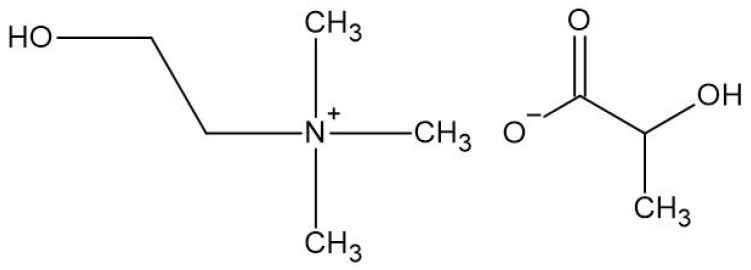
Chemical structures of choline lactate [Chol][LA].

Choline lactate ([Chol][LA]) was synthesized and purified according to the methodology described in Section 4.2, *Materials and Methods—Salt Synthesis and Aqueous Electrolyte Preparation*. The structure of the obtained compound, presented in Figure 1, was confirmed by ^1^H NMR and ^13^C NMR spectroscopy. Additionally, ATR–FTIR analysis was performed, and the thermal properties of the synthesized salt were investigated using TGA and DSC techniques.

The obtained salt was a straw-colored liquid with a viscosity higher than that of water and comparable to that of glycerol. Therefore, it can be classified as an ionic liquid [36,37]. Differential scanning calorimetry (DSC) analysis of choline lactate did not reveal any phase transitions within the investigated temperature range, suggesting that the crystallization temperature of the salt is lower than −70 °C.

**Figure 2:**
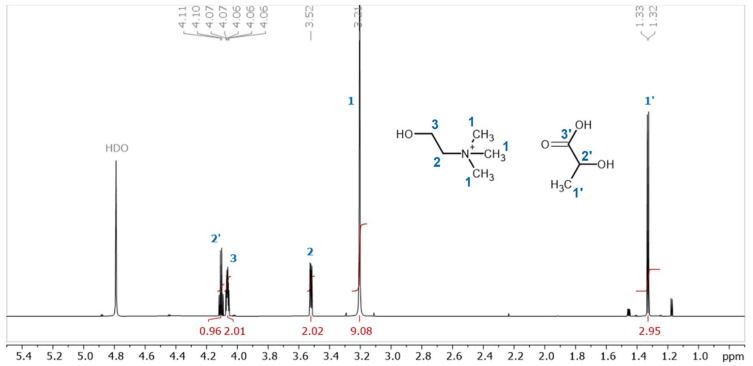
^1^H NMR spectrum of choline lactate (1:1) in D_2_O at 25 °C.

**Figure 3:**
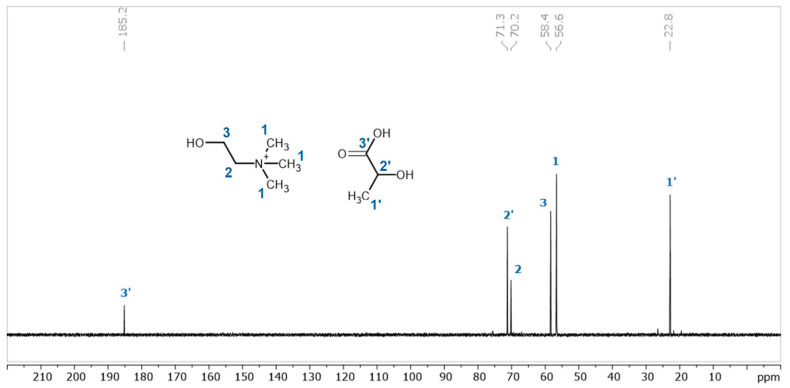
^13^C NMR spectrum of choline lactate (1:1) in D_2_O at 25 °C.

The synthesized choline lactate was determined on the basis of ^1^H NMR (Figure 2) and ^13^C NMR (Figure 3) spectra. The results of the NMR measurements are as follows:

^1^H NMR (800 MHz, D_2_O): δ 4.11 (q, *J* = 6.9 Hz, 1H, H2’), 4.06 (m, 2H, H3), 3.52 (m, 2H, H2), 3.21 (s, 9H, H1), 1.33 (d, *J* = 6.9 Hz, 3H, H1’). ^13^C NMR (201 MHz, D_2_O): δ 185.2 (C3’), 71.3 (C2’), 70.2 (C2), 58.4 (C3), 56.6 (C1), 22.8 (C1’).

**Figure 4:**
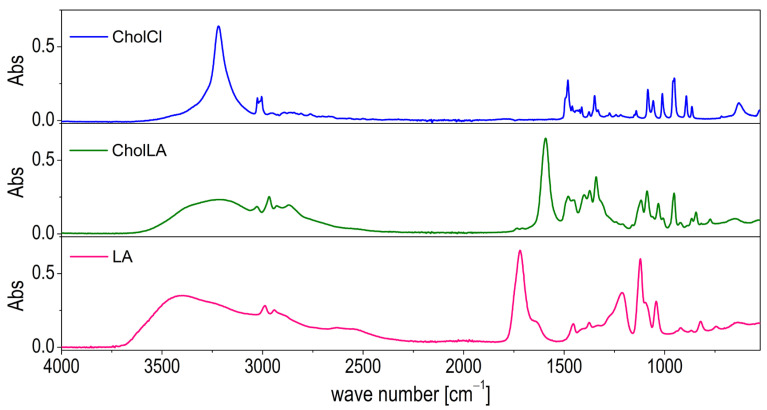
ATR-FTIR spectra of choline chloride (CholCl), lactic acid (LA) and choline lactate ([Chol][LA]).

The successful synthesis of choline lactate was additionally confirmed by ATR–FTIR spectroscopy. Figure 4 presents the spectra of the starting reagents used for choline lactate synthesis, namely choline chloride and lactic acid, together with the spectrum of the obtained choline lactate salt. In the spectrum of choline chloride, characteristic absorption bands corresponding to the choline cation were observed, including a broad O–H stretching band with a maximum at 3219 cm^−1^ and a characteristic quaternary ammonium C–N^+^ stretching band at 952 cm^−1^. The most significant absorption bands in the spectrum of lactic acid are associated with the carboxyl group, which participates in the neutralization reaction during salt synthesis. A broad absorption band in the range of 3600–3000 cm^−1^ was observed and attributed to the stretching vibrations of hydroxyl groups originating from both lactic acid and residual water. The characteristic absorption band of the carboxylic acid carbonyl group appeared at 1720 cm^−1^. Additionally, a shoulder visible in the 1650–1600 cm^−1^ region indicates strong intermolecular interactions associated with hydrogen-bond formation. The spectra of both starting compounds also exhibited characteristic C–H stretching vibrations associated with hydrocarbon groups in the ranges of 3000–2800 cm^−1^ and 1480–1460 cm^−1^. The spectrum of the reaction product displayed a broad absorption band in the 3600–3000 cm^−1^ region, corresponding to O–H stretching vibrations, as well as a characteristic C–N^+^ absorption band with a maximum at 953 cm^−1^, confirming the presence of the choline cation. Furthermore, the absorption band assigned to the carbonyl group shifted to 1557 cm^−1^, indicating the ionic nature of the carboxylate group. These results confirm the successful formation of choline lactate.

**Figure 5:**
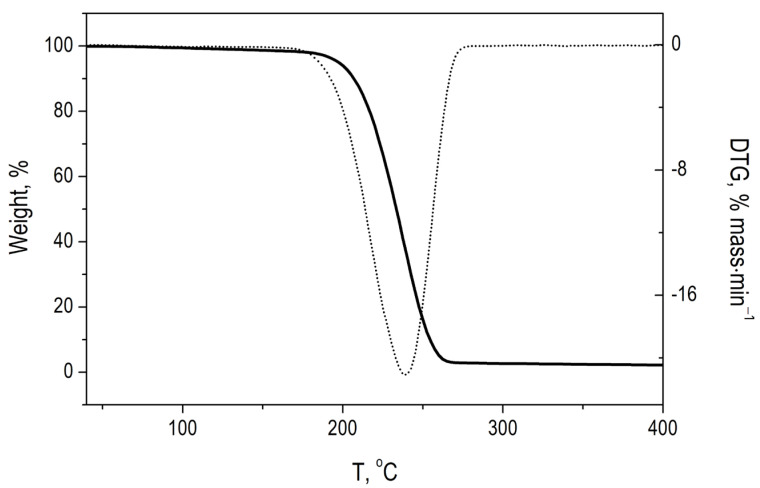
TG (continuous line) and DTG (dashed line) of choline lactate.

Thermogravimetric analysis (TGA) was performed to evaluate the thermal stability of the synthesized salt. As shown in Figure 5, the thermal decomposition of choline lactate occurred in a single step. The temperatures corresponding to 2% and 5% mass loss, commonly regarded as indicators of the thermal stability of the tested compound, were determined to be 179.3 °C and 197.5 °C, respectively. Furthermore, the temperatures corresponding to 10% and 50% mass loss were found to be 206.9 °C (T_10%_) and 233.4 °C (T_50%_), respectively.

#### 2.1.2. Choline Lactate Electrolytes

The synthesized and purified choline lactate salt was used to prepare aqueous solutions at concentrations of 1.0, 1.5, 2.0, 2.5, and 3.0 mol per kg of water. The resulting solutions were then characterized. Their basic physicochemical properties, viscosity and density, were examined at room temperature.

Viscosity and density measurements constituted an essential part of the characterization process, as these parameters provide valuable insight into the potential behavior of aqueous solutions of the synthesized salt in subsequent applications. Viscosity directly influences ion mobility, mass transport, and overall electrochemical performance; therefore, understanding this property is crucial for assessing the suitability of the prepared solutions.

**Figure 6:**
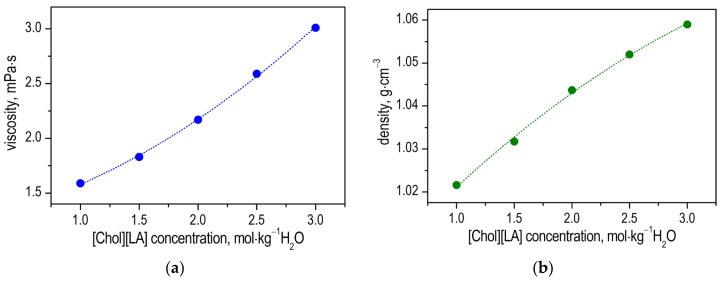
(**a**) Viscosity and (**b**) density of aqueous choline lactate solutions at various concentrations.

The availability of viscosity data for choline-based salts remains relatively limited in the literature, with most studies focusing primarily on aqueous choline chloride systems. Reported viscosity values for choline chloride solutions increase with concentration, reaching approximately 1.07 mPa·s at 0.8113 mol·kg^−1^ and 1.27 mPa·s at 1.7945 mol·kg^−1^, with even higher values observed at greater concentrations [38]. In comparison, the choline lactate solutions investigated in the present study exhibited higher viscosity values (Figure 6). This behavior may be attributed to the presence of the lactate anion, which alters intermolecular interactions within the system and increases resistance to flow. Furthermore, the organic nature of the lactate ion may contribute to the formation of more extensive hydrogen-bonding networks, thereby increasing the viscosity of the solution.

For choline lactate solutions, viscosity increased with increasing concentration. The obtained polynomial fit, η = 1.238 + 0.2174·C + 0.1257·C^2^ (where *C* denotes the concentration of the aqueous choline lactate solution), exhibited a high coefficient of determination (R^2^ = 0.998), indicating excellent agreement between the experimental data and the applied model, as well as high consistency of the measured values.

A similar relationship was observed for the density of aqueous choline lactate solutions, where density increased with increasing solution molality. As in the case of viscosity, the obtained dependence was non-linear and was well described by the equation ρ = 0.9937 + 0.0303·C − 0.0028·C^2^, characterized by a high coefficient of determination (R^2^ = 0.996).

The salt solutions were prepared for potential application as electrolytes in the synthesis of polymer hydrogels intended for use as separators in electrochemical capacitors. Therefore, the next stage of the study involved determining the ionic conductivity of the salt solutions as a function of concentration to find the electrolyte with highest conductivity for application in HPE.

**Figure 7:**
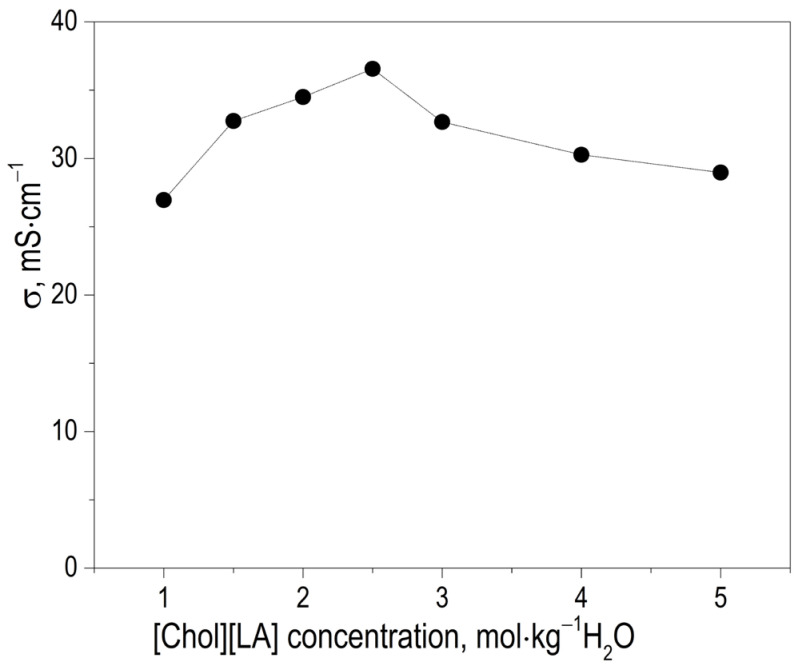
Conductivity of aqueous choline lactate solutions at various concentrations.

Figure 7 presents the dependence of ionic conductivity (σ) on solution concentration. An initial increase in ionic conductivity is observed with increasing concentration, reaching a maximum σ = 36.5 mS·cm^−1^ at approximately 2.5 mol·kg^−1^. Further increases in concentration result in a gradual decrease in ionic conductivity.

The observed behavior suggests that, at lower concentrations, the increase in the number of charge carriers is the dominant factor influencing conductivity. At higher concentrations, however, ion–ion interactions and the increased viscosity of the system likely reduce ionic mobility, leading to a decrease in conductivity despite the higher electrolyte concentration.

### 2.2. Hydrogel Synthesis and Characterization

**Figure 8:**
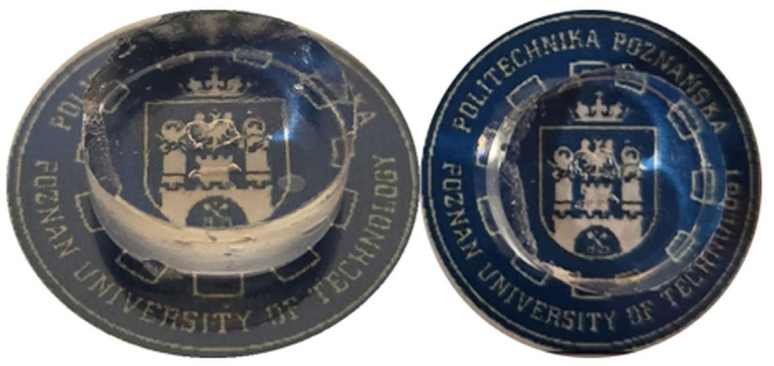
Photographs of the transparent hydrogel polymer electrolyte (HPE) containing 85 wt% aqueous choline lactate solution, demonstrating its homogeneous structure and absence of visible electrolyte leakage.

Based on ionic conductivity measurements of aqueous choline lactate solutions, a solution containing 2.5 mol [Chol][LA] per kilogram of water was selected for hydrogel preparation. Transparent precursor mixtures were prepared using 70, 80, 85, and 90 wt% of the aqueous [Chol][LA] solution and 30, 20, 15, and 10 wt% of the monomer/photoinitiator mixture (Table 1), respectively. As the electrolyte content increased, the concentration of polymerizable species in the precursor mixture decreased, resulting in progressive dilution of the polymer network. To compensate for this effect and preserve the structural integrity of the hydrogels, the content of the tetrafunctional crosslinking monomer TtEGDA was systematically increased. Accordingly, the calculated fraction of polymerizable double bonds originating from TtEGDA (f_TtEGDA_, Table 1) increased from 0.0127 for HPE_70 to 0.1052 for HPE_90, reflecting the increasing theoretical crosslinking potential of the formulations and the design strategy adopted to maintain a stable three-dimensional network despite the increasing electrolyte loading. Photopolymerization of the obtained mixtures yielded flexible and transparent hydrogels polymer electrolytes (HPE) without observable electrolyte leakage. Moreover, no electrolyte syneresis was detected during compression tests or long-term storage. A photograph of the HPE containing 85 wt% electrolyte is presented in Figure 8, illustrating its transparency and homogeneous appearance. The remaining synthesized HPE samples exhibited an identical transparent and homogeneous appearance.

#### 2.2.1. The Degree of Conversion of C=C Double Bonds

In the polymerization process, particularly during in situ hydrogel synthesis in the presence of a solvent, determining the degree of conversion of reactive C=C bonds is essential for obtaining a polymer matrix with an appropriate structure. Therefore, the obtained hydrogels were analyzed by ATR–FTIR spectroscopy after the photopolymerization process to evaluate the conversion of unsaturated C=C bonds.

**Figure 9:**
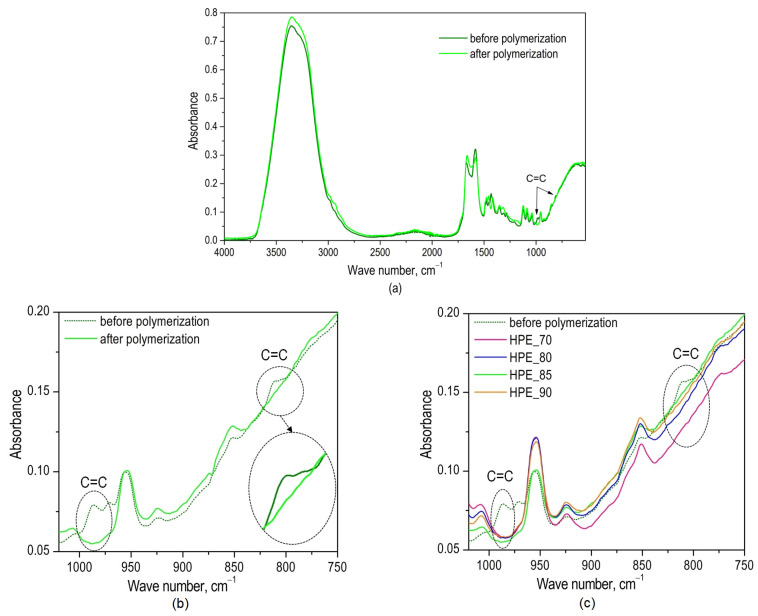
ATR–FTIR spectra of the system containing 85 wt% electrolyte recorded before and after polymerization: (**a**) over the entire investigated wavenumber range, (**b**) in the region corresponding to the absorption bands of unsaturated C=C bonds; (**c**) spectra of hydrogel polymer electrolytes containing different electrolyte concentrations in the region of the absorption bands assigned to unsaturated C=C bonds.

Figure 9a,b presents the spectra recorded before and after polymerization for a selected composition containing 85 wt% aqueous choline lactate solution over the entire investigated wavenumber range, with particular emphasis on the region corresponding to unsaturated bonds. As shown in the spectrum of the precursor mixture before polymerization, the characteristic absorption bands associated with vibrations of unsaturated C=C bonds were observed at 810 cm^−1^ and 987 cm^−1^. In contrast, the absorption band at approximately 1640 cm^−1^, typically assigned to methacrylate and vinyl groups, was overlapped by absorption bands originating from the salt solution. As can be observed from the spectrum recorded for the obtained hydrogel polymer electrolyte, the absorption bands at 810 cm^−1^ and 987 cm^−1^ completely disappeared after the photopolymerization process. This indicates full conversion of the reactive double bonds and confirms the formation of a properly cross-linked polymer network.

Analogous spectra were also recorded for the remaining HPEs containing 70, 80, and 90 wt% aqueous salt solution, and the results are presented in Figure 9c. In all cases, complete disappearance of the absorption bands assigned to C=C bonds was observed after polymerization, indicating full conversion of the reactive double bonds regardless of the composition of the precursor system. Since the polymerization was carried out using a tetrafunctional crosslinking monomer (TtEGDA), the complete consumption of the carbon–carbon double bonds confirms the formation of a three-dimensional crosslinked polymer network. Consequently, the resulting hydrogel polymer electrolytes consist of a covalently crosslinked matrix capable of effectively retaining the liquid electrolyte within its structure. To further confirm the formation of a crosslinked polymer network, the gel fraction of the synthesized hydrogel polymer electrolytes was evaluated by Soxhlet extraction. However, the calculated residual mass after extraction exceeded the theoretical polymer content, yielding apparent gel fraction values above 100%. This observation indicates that, in addition to the crosslinked polymer network, the extracted samples retained compounds that were not completely removed during the extraction procedure. The most plausible explanation is the presence of residual choline lactate strongly confined within the polymer matrix together with tightly bound water molecules, which persisted even after drying under vacuum at 80 °C. Consequently, the gel fraction values obtained by this method should not be interpreted as an accurate measure of the insoluble polymer content. Nevertheless, the presence of a substantial insoluble residue following prolonged Soxhlet extraction provides clear evidence for the successful formation of a covalently crosslinked three-dimensional polymer network.

#### 2.2.2. Thermal Characterization

When aqueous salt solutions are used as electrolytes, a major limitation is their narrow operating temperature window, particularly at low temperatures, due to water crystallization. In the crystalline structure of ice, ion mobility is significantly restricted, preventing effective ionic conduction. Numerous approaches have therefore been explored to overcome this limitation, including the application of novel salts and the use of small amounts of additives that inhibit the crystallization of water molecules.

**Figure 10:**
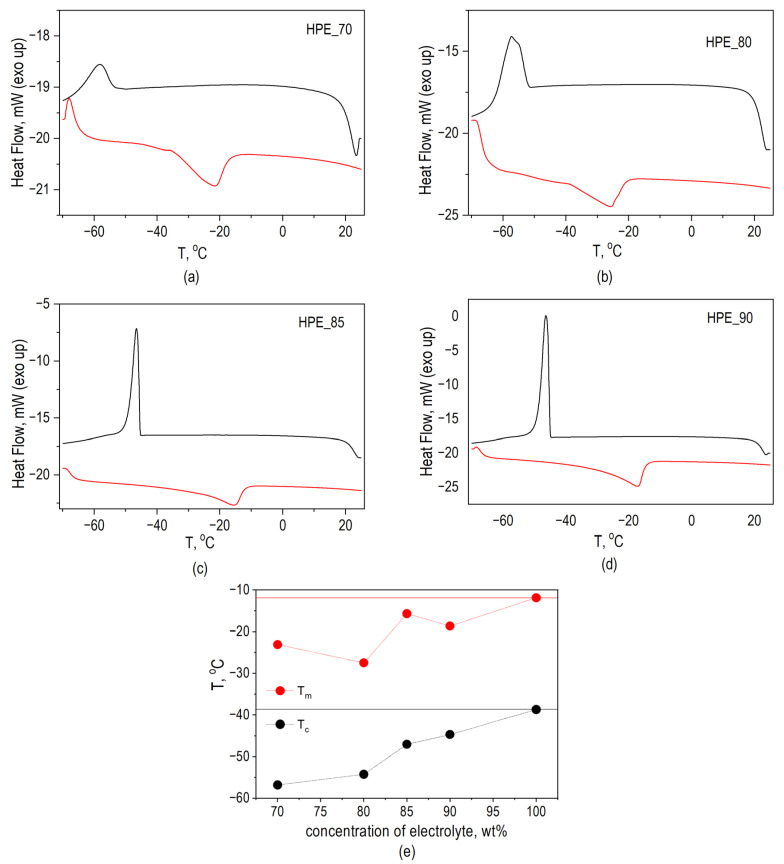
DSC thermograms of the prepared hydrogels containing different amounts of electrolyte (**a**) 70 wt%, (**b**) 80 wt%, (**c**) 85 wt%, and (**d**) 90 wt%, and (**e**) dependence of crystallization temperature (T_c_) and melting temperature (T_m_) on the electrolyte content in hydrogels.

Differential scanning calorimetry (DSC) measurements of phase transitions were performed, and the corresponding thermograms are presented in Figure 10. The salt investigated in this study not only represents a green electrolyte component but also significantly decreases the crystallization temperature of a solution containing 2.5 M salt per kilogram of water to −38.7 °C. This result is particularly significant because, in the hydrogel systems, the crystallization temperature was reduced even further. Hydrogels containing from 70 to 90 wt% of the 2.5 mol·kg^−1^ aqueous choline lactate solution exhibited electrolyte crystallization temperatures below −44 °C. Moreover, the crystallization temperature shifted toward lower values with increasing polymer matrix content in the hydrogel, reaching −56.8 °C for the hydrogel containing 70 wt% electrolyte. In addition, the melting temperatures observed for the hydrogel systems were also lower than that of the pure electrolyte solution and remained below −15 °C, reaching the lowest value for the hydrogel containing 80 wt% electrolyte, i.e., −27.5 °C. The lower phase transition temperatures observed for the electrolyte confined within the HPE structures, relative to the pure electrolyte, can be attributed to restricted structural ordering of the electrolyte molecules within the polymer matrix network.

A slight deviation from the observed non-linear trend in the phase-transition temperatures, namely the crystallization and melting temperatures, was noted for the HPE containing 85 wt% electrolyte. This behavior may be attributed to a lower cross-linking density than in the other hydrogels, in which the proportion of the cross-linking monomer TtEGDA relative to the non-cross-linking monomer AAm was higher. The earlier crystallization and delayed melting observed in this sample are likely associated with increased electrolyte mobility within the less densely cross-linked HPE matrix.

#### 2.2.3. Mechanical Characterization of HPE by Compression Testing

**Figure 11:**
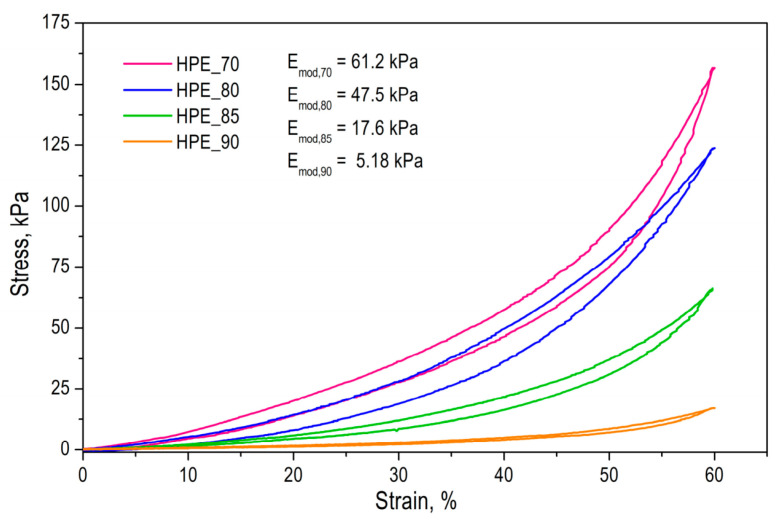
Stress–strain curves obtained from compression tests of hydrogel polymer electrolytes (HPE) with different compositions (70 wt%, 80 wt%, 85 wt%, and 90 wt% of electrolyte).

Figure 11 presents the stress–strain curves obtained during compression testing of four hydrogel polymer electrolytes with different compositions (70 wt%, 80 wt%, 85 wt%, and 90 wt% of electrolyte). All samples exhibited a characteristic non-linear mechanical response typical of soft hydrogel materials, with stress increasing progressively with increasing strain. At low deformations, the materials showed an approximately linear elastic region, followed by gradual stiffening at higher strain values.

Among the tested samples, the HPE_70 exhibited the highest mechanical resistance to compression, reaching stress values above c.a. 150 kPa at 60% strain. This sample also showed the highest Young’s modulus (61.2 kPa), reflecting the highest polymer content in the hydrogel structure and, consequently, the greatest resistance to mechanical deformation among the investigated materials. The 80 wt% hydrogel displayed slightly lower stiffness and compressive strength, with a Young’s modulus of 47.5 kPa, while maintaining a similar overall deformation profile.

**Figure 12:**
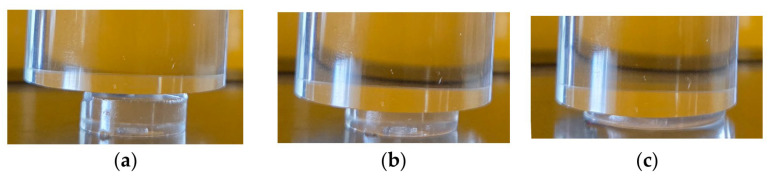
Photographs of the hydrogel polymer electrolyte (HPE) containing 85 wt% electrolyte: (**a**) before compression testing, (**b**) at 60% compressive strain, and (**c**) at 80% compressive strain.

A substantial decrease in mechanical properties was observed for the 85 wt% and 90 wt% hydrogels. The sample HPE_85 reached approximately 65 kPa at 60% strain and exhibited a Young’s modulus of 17.6 kPa, suggesting a significantly weaker polymer structure. The sample HPE_90 demonstrated the softest behavior among all tested materials, with stress values remaining below 20 kPa over the entire deformation range and a Young’s modulus of only 5.18 kPa. The markedly lower stiffness of this sample indicates a highly flexible and loosely crosslinked network. In addition, photographs of the HPE containing 85 wt% electrolyte before compression testing and at 60% and 80% compressive strain are presented in Figure 12. The images demonstrate the excellent deformability of the hydrogel and, importantly, show no visible electrolyte syneresis even under high compressive deformation.

The results demonstrate a clear dependence of the mechanical properties on hydrogel composition. Increasing the electrolyte percentage in HPE from 70 wt% to 90 wt% resulted in a progressive reduction in compressive strength and elastic modulus, indicating decreased rigidity and reduced resistance to deformation.

#### 2.2.4. Conductivity of Hydrogel Polymer Electrolytes

**Figure 13:**
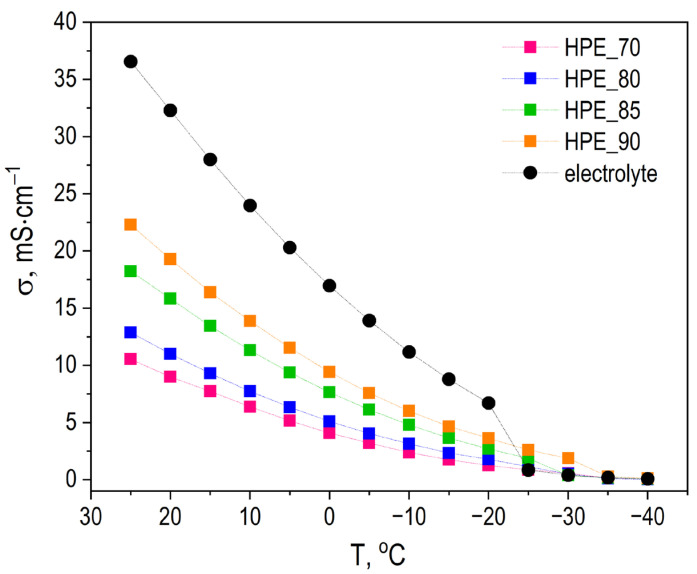
Dependencies of ionic conductivity on temperature for HPE with different content of electrolyte and for pure electrolyte (2.5 mol [Chol][LA] per kg H_2_O).

Figure 13 shows the temperature dependence of ionic conductivity for the HPE samples containing different amounts of electrolyte (70–90 wt%) and for the pure electrolyte (2.5 mol·kg^−1^ [Chol][LA]). In all investigated systems, the ionic conductivity increased with increasing temperature. The highest conductivity values were observed for the pure electrolyte, reaching 36.6 mS·cm^−1^ at 298 K, whereas the HPE samples exhibited lower conductivities due to the presence of the polymer matrix restricting electrolyte ion mobility. Nevertheless, increasing the electrolyte content in the HPE resulted in a systematic enhancement of conductivity, from 10.5 mS·cm^−1^ for HPE_70 to 22.3 mS·cm^−1^ for HPE_90 at 298 K. This behavior confirms that a higher electrolyte fraction facilitates ion transport through the polymer network. The results also show that the liquid electrolyte freezes at temperatures below −20 °C, which results in a significant decrease in ionic conductivity. Hydrogel polymer electrolytes have a shift in the crystallization temperature of the liquid phase toward lower temperatures by approximately 5–10 °C. These results are consistent with a similar trend observed in DSC measurements.

**Figure 14:**
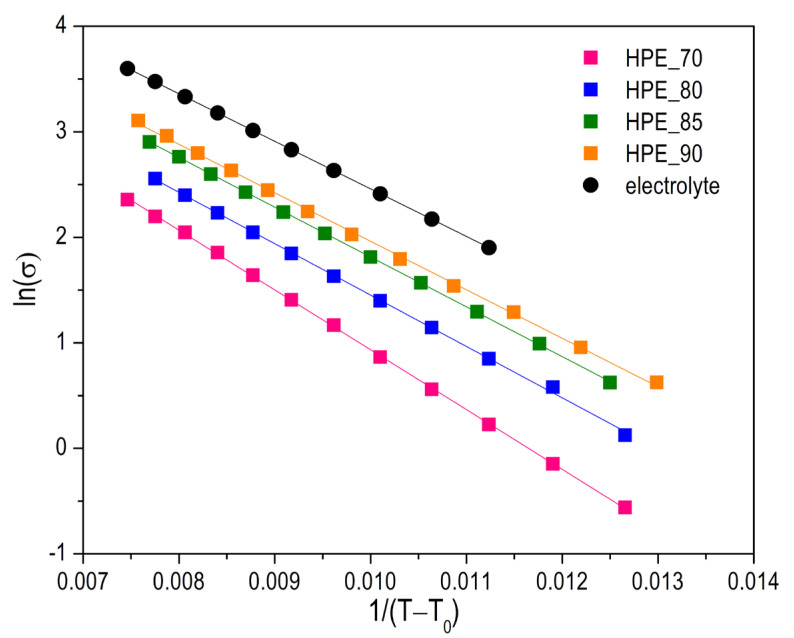
The dependence of ln(σ) on 1/(T − T_0_) for HPE with different content of electrolyte and for pure electrolyte (2.5 M [Chol][LA] per kg H_2_O). Experimental data were fitted by linear regression based on the VTF equation.

The conductivity data were analyzed using the Vogel–Tammann–Fulcher (VTF) model, which is commonly applied to amorphous polymer electrolytes where ion transport is coupled with segmental motion of polymer chains. The dependence of ln(σ) versus 1/(T − T_0_) is presented in Figure 14. The excellent linearity of the plots (R^2^ > 0.999 for all samples) demonstrates that the conductivity behavior follows the VTF relationship (1).

(1) $$\sigma =\sigma_{\infty }exp\left(-\frac{B}{T-T_{0}}\right)$$

where σ_∞_ is the pre-exponential factor, B is the pseudo-activation parameter, and T_0_ is Vogel temperature (or ideal glass transition temperature).

The calculated VTF parameters are summarized in Table 2. The T_0_ values for all systems were found in the range of 164–169 K, suggesting similar dynamic behavior of the polymer/electrolyte systems. At the same time, the pseudo-activation energy (E_a,VTF_) decreased gradually with increasing electrolyte content, from 4.71 kJ·mol^−1^ for HPE 70% to 3.82 kJ·mol^−1^ for HPE_90, while the pure electrolyte exhibited the lowest value of 3.74 kJ·mol^−1^. The decrease in activation energy indicates facilitated ion migration in systems with higher electrolyte content, which is consistent with the observed increase in ionic conductivity. Furthermore, the relatively high σ_∞_ values suggest a significant concentration of mobile ionic species in all investigated samples.

Overall, the obtained results demonstrate that the incorporation of larger amounts of electrolyte into the HPE matrix effectively improves ion transport properties while maintaining VTF-type conduction behavior characteristic of amorphous polymer electrolyte systems.

### 2.3. Electrochemical Investigation of Supercapacitor

**Figure 15:**
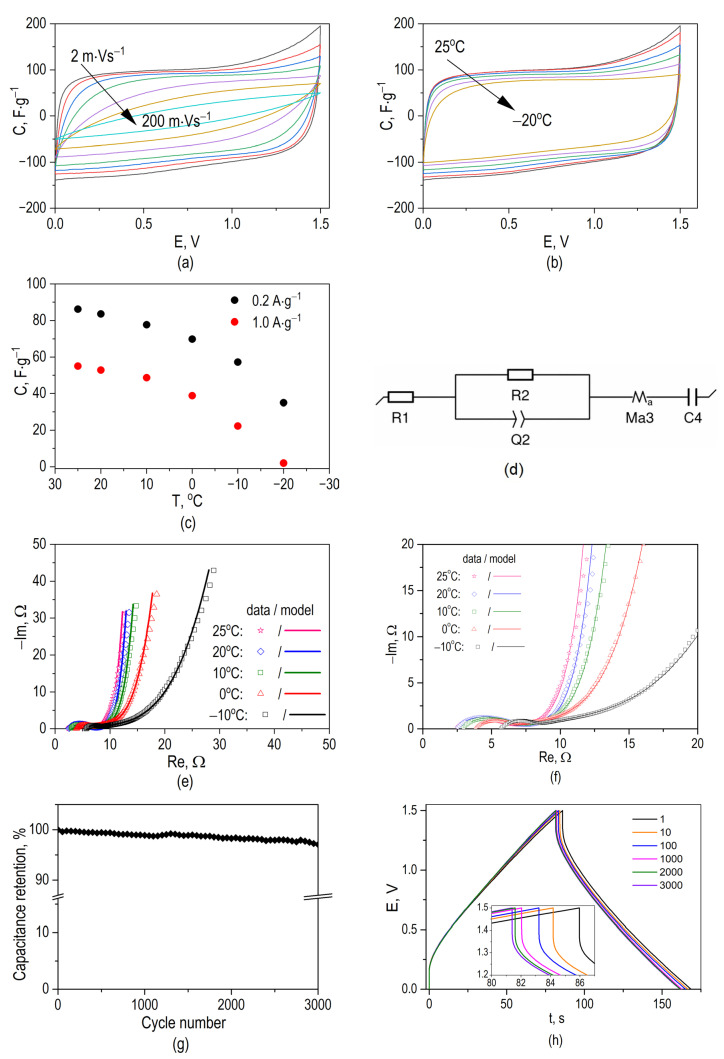
Electrochemical performance of the supercapacitor investigated in the temperature range from 25 °C to −20 °C: (**a**) cyclic voltammograms recorded at different temperatures (scan rate 2 mV·s^−1^), (**b**) specific capacitance as a function of temperature at current densities of 0.2 and 1.0 A·g^−1^, (**c**) cyclic voltammograms recorded at scan rates from 2 to 200 mV·s^−1^ at 25 °C, and (**d**) equivalent circuit model (**e**,**f**) Nyquist plots obtained at different temperatures: experimental results and equivalent circuit model fitting, (**g**) cycling stability (to enhance readability, the plot shows every 50th point), (**h**) Galvanostatic charge–discharge (GCD) curves recorded during cycling stability measurements; numbers indicate cycle number.

Figure 15 presents the electrochemical performance of the investigated supercapacitor in the temperature range from 25 °C to −20 °C using cyclic voltammetry, galvanostatic charge–discharge measurements, and electrochemical impedance spectroscopy. The obtained results demonstrate a clear influence of temperature on the capacitive behavior and charge-transfer properties of the device.

The scan-rate-dependent CV measurements (Figure 15a) revealed increasing polarization effects at higher scan rates, while the overall capacitive response remained preserved. This behavior confirms good rate capability and structural stability of the electrode material despite kinetic limitations at fast charging/discharging conditions.

The cyclic voltammograms (Figure 15b) retained a quasi-rectangular shape over the entire temperature range, indicating that the supercapacitor preserved its capacitive characteristics even at subzero temperatures. However, a gradual decrease in the enclosed CV area was observed with decreasing temperature, confirming a reduction in charge storage capability caused by lower electrolyte ionic conductivity and slower ion diffusion.

The specific capacitance systematically decreased with decreasing temperature at both investigated current densities (Figure 15c). At 0.2 A·g^−1^, the capacitance decreased from approximately 86 F·g^−1^ at 25 °C to about 35 F·g^−1^ at −20 °C. A similar trend was observed at 1.0 A·g^−1^, where the capacitance dropped from around 55 F·g^−1^ to nearly 2 F·g^−1^ at −20 °C. These results indicate that low temperatures significantly limit ion transport and reduce the electrochemical accessibility of the electrode surface, particularly at higher current densities.

Electrochemical impedance spectroscopy (EIS), fitted using an equivalent circuit model (Figure 15d), showed a substantial increase in internal resistance with decreasing temperature (Figure 15e,f). The Nyquist plots exhibited a progressive shift toward higher real impedance values and steeper low-frequency regions at lower temperatures, indicating increased electrolyte resistance and hindered ion diffusion processes. Nevertheless, the supercapacitor remained electrochemically active throughout the investigated temperature range.

Electrochemical impedance spectra recorded between 25 and −10 °C were fitted using the equivalent circuit R_1_ + (R_2_||Q_2_) + Ma + C, where R_1_ represents the equivalent series resistance (ESR), (R_2_||Q_2_) describes the high-frequency interfacial response, Ma is a modified transmission-line element accounting for distributed ion transport within the porous activated carbon electrode, and C is an additional capacitive contribution required to reproduce the low-frequency behavior.

The selected equivalent circuit provided satisfactory agreement with the experimental impedance spectra over the entire investigated temperature range. The fitting procedure resulted in acceptable parameter uncertainties indicating that the proposed model adequately describes the electrochemical response of the EDLC under different operating temperatures (Table 3).

**Table 3:** Fitted equivalent circuit parameters obtained from EIS analysis.

Parameter	25 °C	20 °C	10 °C	0 °C	−10 °C
R_1_ (Ω)	1.70	1.76	2.16	3.29	5.23
R_2_ (Ω)	3.19	3.62	2.86	1.66	1.45
Q_2_ (F·s^α−1^)	5.2 × 10^−5^	4.43 × 10^−5^	3.29 × 10^−5^	5.9 × 10^−6^	2.3 × 10^−6^
α_2_	0.77	0.70	0.74	0.91	0.97
Rd (Ω)	7.67	8.74	10.2	11.9	16.4
τ (s)	66	111	87.5	29.2	10.6
α_Ma_	0.24	0.26	0.26	0.29	0.35
C (F)	0.54	0.53	0.51	0.49	0.48

The equivalent series resistance (R_1_) increased from 1.70 Ω at 25 °C to 5.23 Ω at −10 °C, indicating a progressive increase in the internal resistance of the EDLC caused by the reduced ionic conductivity of the gel electrolyte at lower temperatures. The distributed pore resistance (Rd) increased monotonically from 7.67 Ω to 16.4 Ω, confirming that ion transport within the porous activated carbon network became increasingly hindered as the temperature decreased. The parameter Q_2_ decreased significantly with decreasing temperature, indicating a reduction in the effective capacitive response probably due to limited accessibility of the porous electrode surface. Meanwhile, the exponent α_2_ remained within the range of 0.70–0.97, suggesting predominantly capacitive behavior of the high-frequency process. The additional capacitor C remained nearly constant (0.48–0.54 F), indicating a stable capacitive contribution throughout the investigated temperature range. The transmission-line exponent αMa (0.24–0.35) reflects a broad distribution of relaxation times associated with the heterogeneous pore structure and distributed ion transport. Although the transmission-line time constant τ did not exhibit a monotonic temperature dependence, it should be interpreted cautiously because it is strongly coupled with the remaining transmission-line parameters.

The equivalent circuit analysis indicates that the deterioration of EDLC performance at low temperatures is primarily governed by increasing ionic transport limitations. The monotonic increase in both the equivalent series resistance (R_1_) and the distributed pore resistance (Rd) demonstrates the progressive reduction of ionic conductivity in the gel electrolyte and slower ion migration within the porous activated carbon electrode. The substantial decrease in Q_2_ suggests that a smaller fraction of the porous surface remains electrochemically accessible at low temperatures, whereas the nearly constant value of C indicates that this parameter represents a stable capacitive contribution of the system.

The cyclic stability of the capacitor was evaluated by performing 3000 galvanostatic charge–discharge cycles at −10 °C (Figure 15g). The device exhibited excellent electrochemical stability under low-temperature conditions, retaining approximately 97% of its initial discharge capacitance after 3000 cycles. The capacitance loss was limited to only 3%, indicating a high degree of reversibility and remarkable long-term durability of the electrode–electrolyte system. These results demonstrate that the investigated capacitor maintains stable performance even under subzero operating conditions, highlighting its potential for low-temperature energy storage applications.

Galvanostatic charge–discharge (GCD) curves recorded during cycling stability measurements are presented in Figure 15h. The equivalent series resistance (ESR) was calculated from the instantaneous voltage drop (IR drop) at the charge/discharge transition according to Equation (2).

(2) $$ESR=\frac{∆V}{2I}$$

where ΔV is the voltage drop and *I* is the applied current.

The IR drop remained nearly constant throughout cycling, changing only from 90 mV in the first cycle to approximately 81–84 mV after 3000 cycles. Accordingly, the calculated ESR decreased slightly from 4.00 Ω to 3.60–3.73 Ω, corresponding to an overall variation of only about 10%. This small change indicates excellent stability of the internal resistance during prolonged cycling. Although the discharge time decreased slightly during the initial stage, the nearly constant ESR suggests that the electronic and ionic conduction pathways remained essentially unchanged. Therefore, the reduction in discharge time is more likely associated with a slight loss of effective capacitance caused by changes in ion accessibility within the porous electrode rather than degradation of the cell resistance. The stabilization of both the discharge profiles and ESR after approximately 100 cycles confirms the excellent electrochemical stability of the fabricated EDLC. Overall, the cycling results demonstrate that the activated carbon/gel electrolyte supercapacitor exhibits high electrochemical durability, with only minor changes in capacitance and internal resistance after 3000 charge–discharge cycles, confirming the good long-term stability of the developed system.

Overall, the studied supercapacitor demonstrates stable electrochemical operation from 25 °C to −20 °C, although with reduced capacitance and increased resistance at lower temperatures. The results confirm the suitability of the system for low-temperature energy storage applications, while also indicating that further improvements in electrolyte composition and ion transport properties are necessary to enhance electrochemical performance under subzero operating conditions.

## 3. Conclusions

In this work, sustainable hydrogel polymer electrolytes based on aqueous choline lactate were successfully prepared and evaluated as separator–electrolyte systems for low-temperature supercapacitors. The synthesized choline lactate was successfully characterized by NMR, ATR–FTIR, DSC, and TGA analyses and showed good thermal stability, with 2% and 5% mass loss temperatures of 179.3 °C and 197.5 °C, respectively. Among the investigated aqueous electrolytes, the solution containing 2.5 mol of choline lactate per kilogram of water showed the highest ionic conductivity of 36.5 mS·cm^−1^ and was selected for hydrogel preparation.

Photopolymerization yielded transparent, homogeneous, and mechanically stable hydrogels with excellent electrolyte retention. ATR–FTIR analysis confirmed complete conversion of the reactive C=C double bonds, indicating successful formation of a crosslinked polymer network. The hydrogels showed a pronounced anti-freezing effect. Compared with the pure electrolyte, which crystallized at −38.7 °C, the crystallization temperatures of the hydrogel systems were shifted below −44 °C and reached −56.8 °C for HPE_70. This confirms that confinement of the choline lactate electrolyte within the polymer matrix effectively suppresses water crystallization and extends the low-temperature stability of the system.

The composition of the hydrogels strongly influenced their mechanical and transport properties. Increasing the electrolyte content from 70 to 90 wt% enhanced ionic conductivity from 10.5 to 22.3 mS·cm^−1^ at 25 °C and decreased the VTF pseudo-activation energy from 4.71 to 3.82 kJ·mol^−1^, indicating improved ion mobility. At the same time, Young’s modulus decreased from 61.2 to 5.18 kPa, showing that higher electrolyte loading produced softer and more flexible hydrogels. Despite this decrease in stiffness, the hydrogels maintained structural integrity and no electrolyte syneresis was observed during compression testing.

Electrochemical measurements confirmed that the assembled supercapacitor retained capacitive behavior over the investigated temperature range from 25 °C to −20 °C. At 0.2 A·g^−1^, the specific capacitance decreased from approximately 86 F·g^−1^ at 25 °C to about 35 F·g^−1^ at −20 °C, while impedance measurements showed increased internal resistance at lower temperatures. These changes are associated with reduced ionic conductivity and slower ion diffusion under subzero conditions. Nevertheless, the device remained electrochemically active throughout the tested temperature range. Cycling tests performed at −10 °C further demonstrated excellent durability, with approximately 97% capacitance retention after 3000 charge–discharge cycles and only minor changes in the equivalent series resistance, confirming the high stability of both the hydrogel electrolyte and the electrode–electrolyte interface during prolonged low-temperature operation.

Equivalent circuit analysis revealed that the deterioration of electrochemical performance at low temperatures is primarily governed by increasing ionic transport limitations. The monotonic increase in both the equivalent series resistance and the distributed pore resistance demonstrates reduced ionic conductivity of the hydrogel electrolyte and hindered ion migration within the porous activated carbon electrode. In contrast, the nearly constant value of the additional capacitive element suggests that the intrinsic capacitive contribution of the device remains largely unaffected by decreasing temperature. Overall, the obtained results demonstrate that choline lactate-based hydrogel polymer electrolytes combine environmental sustainability, good electrolyte retention, anti-freezing properties, ionic conductivity, and mechanical flexibility. Therefore, they represent promising materials for flexible and environmentally friendly supercapacitors intended for low-temperature energy-storage applications. Although the present study demonstrated promising results, electrochemical tests were limited to temperatures down to −20 °C and cycling stability was evaluated only at −10 °C. Future work will focus on extending electrochemical evaluation to lower temperatures and further optimizing the electrolyte composition to improve subzero performance.

## 4. Materials and Methods

### 4.1. Materials

Monomers acrylamide (AAm, purity ≥ 99%), tetraethyleneglycol diacrylate (TtEGDA), and choline chloride (purity ≥ 98%) were purchased from Sigma-Aldrich (St. Louis, MO, USA). Photoinitiator 1-hydroxycyclohexyl phenyl ketone (HCPK, Irgacure 184) was received from BASF (Ludwigshafen, Germany). Sodium hydroxide (purity ≥ 98.8%) and lactic acid (water solution, ≥88–92%) were purchased from Chempur (Piekary Śląskie, Poland). All chemicals were used without further purification.

### 4.2. Salt Synthesis and Aqueous Electrolyte Preparation

**Figure 16:**
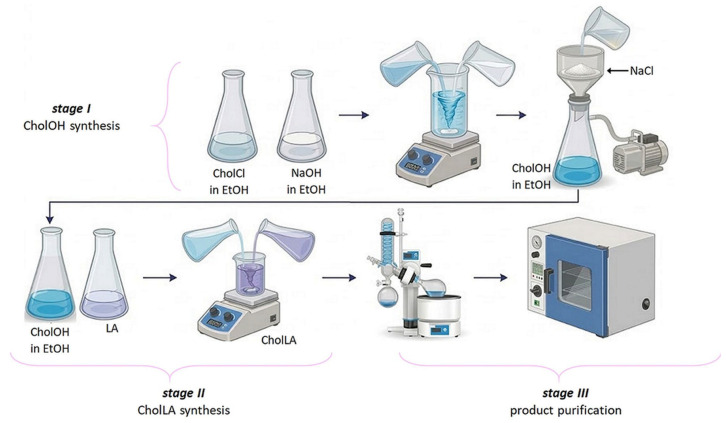
Scheme of choline lactate synthesis and purification.

The process of synthesizing high-purity choline lactate consists of three main steps. The first involves the synthesis of choline hydroxide from choline chloride. The second step involves obtaining choline lactate from the main components, prepared choline hydroxide and lactic acid. The final step involves purifying the resulting salt. The synthesis and purification procedure of choline lactate is schematically illustrated in Figure 16.

Step one: The method for preparing choline hydroxide is based on an ion-exchange reaction between choline chloride and sodium hydroxide. Choline chloride and sodium hydroxide were used in a 1:1 equimolar ratio. The appropriate amount of reagents was dissolved in ethanol and stirred on a magnetic stirrer (DLAB, MS-H-Pro*, Beijing, China) for approximately 1 h at room temperature (≈20 °C). Ethanol was chosen for the synthesis because choline hydroxide dissolves in it, and sodium chloride, a by-product of the reaction, precipitates. To remove the undesirable sodium salt, the resulting mixture was vacuum filtered, yielding a homogeneous ethanolic solution of choline hydroxide. To prevent possible degradation of the choline hydroxide, the solution was stored in a refrigerator (≈3 °C).

**Figure 17:**
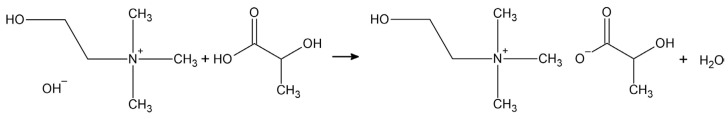
Choline lactate ([Chol][LA]) synthesis reaction.

Step two: Synthesis of choline lactate by acid neutralization with base. Appropriate amounts of lactic acid and ethanolic choline hydroxide solution were weighed to obtain a 1:1 molar ratio of the reactants. The measured amount of lactic acid was added directly to the previously prepared (step one) choline hydroxide solution. The reaction mixture was then placed on a magnetic stirrer and allowed to react at room temperature (≈20 °C) with constant stirring until a clear and homogeneous solution was obtained. Choline salt and water as a reaction byproduct were obtained (Figure 17).

Step Three: Product Purification. After choline lactate synthesis, ethanol was evaporated from the reaction mixture under reduced pressure (approximately 70 mPa) in a rotary evaporator (Rotavapor R-205, BÜCHI Labortechnik AG, Flawil, Switzerland) at 60 °C, followed by drying in a vacuum oven (BINDER GmbH, Tuttlingen, Germany) at 100 °C. The salt was then washed with a mixture of acetone and isopropanol for purification. Due to its hygroscopic nature, the salt was stored in tightly closed containers.

Aqueous electrolytes were prepared by adding appropriate amounts of prepared choline lactate to distilled water to prepare solutions containing 1.0, 1.5, 2.0, 2.5, and 3.0 mol of salt per kilogram of water. The weighed components were mixed in tightly sealed glass vessels at room temperature on a magnetic stirrer until homogeneous solutions were obtained. The prepared solutions were then stored in a refrigerator.

### 4.3. Preparation of Hydrogels

Hydrogels were prepared by in situ photopolymerization of monomers mixture in aqueous electrolyte solution. The electrolyte solution contains 2.5 mol·kg^−1^ H_2_O of choline lactate and constitutes 70, 80, 85 or 90% of the mixture mass, respectively. The monomer mixtures contain appropriate amounts of acrylamide and tetraethyleneglycol diacrylate. Additionally, 0.2% of photoinitiator was added to the mixture as a photoinitiator, relative to the total weight of the composition. The mixtures composed of monomers, aqueous electrolyte, and photoinitiator were homogenized using a digital orbital shaker (MS3, IKA, Staufen, Germany) at 3000 rpm to obtain a uniform, transparent mixture, and subsequently poured into molds. Polymerization was carried out under UV irradiation using an ASN-36W lamp (λ_max_ = 365 nm, light intensity I_0_ = 6 mW·cm^−2^, Asia Nail Beauty Commodity Co., Ltd., Yueqing, China) for 10 min on each side of the mold. After the polymerization process, transparent hydrogels were obtained. The compositions of the photocurable mixtures, together with the corresponding sample designations, are presented in Table 1.

### 4.4. Sample Characterization

#### 4.4.1. Thermal Characteristics

The thermogravimetric measurements were performed using a TG 209 F3 Tarsus analyzer (NETZSCH—Geratebau GmbH, Selb, Germany). Approximately 10 mg of the choline lactate sample was placed in a platinum crucible and analyzed in the temperature range from 30 °C to 600 °C with a heating rate of 10 °C⋅min^−1^ under a nitrogen atmosphere (purge gas flow of 30 mL·min^−1^ and protective gas flow of 20 mL·min^−1^).

Thermal analysis was performed using a PerkinElmer DSC6000 differential scanning calorimeter (PerkinElmer, Waltham, MA, USA). Measurements were performed for [Chol][LA] salt, electrolyte and hydrogels in the temperature range from –70 °C to 25 °C, at a heating rate of 5 °C·min^–1^, in an argon atmosphere (20 mL·min^–1^). The melting (T_m_) and crystallization (T_c_) temperatures were determined from the obtained thermograms.

#### 4.4.2. Spectroscopic Measurements

Infrared (IR) spectra were performed on a Nexus Nicolet 5700 Fourier Transform Infrared Spectrophotometer (FTIR, Thermo Electron Scientific Instruments Corporation, Madison, WI, USA) equipped with an attenuated total reflection (ATR) accessory with a diamond crystal (T = 25 °C, range 4000–525 cm^−1^, resolution 4 cm^−1^ at 32 scans).

The NMR measurements were performed at 25 °C on an Agilent DD2 800 spectrometer (Agilent Technologies, Santa Clara, CA, USA) using a 5 mm ^1^H{^13^C/^15^N} probe. The spectra were acquired in D_2_O and referenced indirectly to an external DSS (sodium 3-(trimethylsilyl)propane-1-sulfonate). The ^1^H NMR data were collected with 32 transients into 64k data points over a spectral width of 12 ppm. A relaxation delay of 10 s was applied between transients. The ^13^C NMR experiment was recorded using a standard one-pulse sequence with broadband proton decoupling. All data were acquired using VnmrJ 4.2 software, processed and analyzed with MestReNova 15.

#### 4.4.3. Physicochemical Characterization (Density and Viscosity)

The density of the tested aqueous salt solutions was measured at room temperature using a Densito 30PX density measuring device (Mettler Toledo, Greifensee, Switzerland).

Viscosity was measured on a Brookfield DV-II+ Pro cone-and-plate apparatus at a temperature of 25 °C. To maintain a constant measurement temperature, the device was equipped with a thermostat Lauda E 100 (LAUDA DR. R. Wobser GmbH & Co. KG, Lauda-Königshofen, Germany). The measurement was performed at a cone rotational speed of 100 rpm.

#### 4.4.4. Mechanical Studies

Hydrogel samples were subjected to compression testing using a CT3 texture analyzer (AMETEK Brookfield, Middleboro, MA, USA). Prior to analysis, cylindrical samples with an approximate thickness of 5 mm and diameter of 10 mm were prepared by photopolymerization in mold. The measurements were carried out in compression mode under displacement control. The test parameters were as follows: test speed of 0.30 mm·s^−1^, return speed of 0.30 mm·s^−1^, trigger load of 0.02 N, and target displacement of 3.0 mm (60%). Two compression cycles were performed without a holding time at maximum deformation.

During the test, force–displacement data were recorded and subsequently converted into stress–strain curves. Stress values were calculated as the ratio of the applied force to the initial cross-sectional area of the sample, while strain was determined as the ratio of sample deformation to the initial sample height. Young’s modulus was determined from the linear region of the stress–strain relationship corresponding to 1–5% strain. The modulus value was calculated as the slope of the linear regression fitted to the data within this deformation range.

#### 4.4.5. Electrochemical Studies

The ionic conductivity (*σ*) was investigated by electrochemical impedance spectroscopy (EIS) in the frequency range from 10 mHz to 1 MHz using the SP-300 potentiostat/galvanostat (Biologic, Seyssinet-Pariset, France). The hydrogel samples, with a surface area of 0.9 cm^2^ and a thickness of 0.5 cm, were used in the as-prepared state. The measurements were conducted in temperature range from (–40) °C to 25 °C in Dynamic climate chamber MK56 (BINDER GmbH, Tuttlingen, Germany) with the use of a two-electrode Swagelok^®^ type electrochemical vessel equipped with 316L stainless steel current collectors and spacer. Before measuring the ionic conductivity, the sample was stabilized for 2 h at a given temperature.

The ionic conductivity was calculated from Equation (3):

(3) $$\sigma =\sigma_{s}\cdot \frac{l}{A}$$

where *σ* is the ionic conductivity of the measured sample in S⋅cm^−1^, *l* is the thickness in cm, *A* is the area in cm^2^, and *σ_S_* represents the volumetric conductance of the sample in S.

Electrochemical performance of the supercapacitors was evaluated in a two-electrode Swagelok^®^ cell using an SP-300 potentiostat/galvanostat (Biologic Science Instruments, Seyssinet-Pariset, France). Prior to cell assembly, carbon electrodes were attached to current collectors. The average mass of activated carbon in a single electrode was approximately 8 mg and was used for the calculation of current density and specific capacitance. The hydrogel membrane was placed between two activated carbon electrodes and used simultaneously as separator and electrolyte reservoir. Electrochemical characterization included cyclic voltammetry (CV) performed at scan rates ranging from 2 to 200 mV⋅s^−1^ within a cell voltage window of 1.5 V, galvanostatic charge/discharge measurements with potential limitation (GCPL) at current densities from 0.2 to 4 A·g^−1^, and electrochemical impedance spectroscopy (EIS) carried out at open-circuit voltage in the frequency range from 1 MHz to 10 mHz with an amplitude of 10 mV. All electrochemical measurements were conducted in the temperature range from (−20) °C to 25 °C; the cells were equilibrated at each temperature for 2 h.

The specific capacitance (F∙g^−1^) was calculated according to Equation (4) [39]:

(4) $$C_{el.int/D}=\frac{2\cdot I\cdot E_{int/D}}{0.5\cdot m_{el}\cdot {∆U}^{2}}$$

where I is the applied current (A), m_el_ (g) is the average mass of activated carbon in electrode, ΔU is the change in cell potential (V), and E_int/D_ is discharge energy calculated by integrating, i.e., finding the area under the galvanostatic discharge curve.

As part of the study, the electrochemical stability of the capacitor was also evaluated. For this purpose, cyclic charge–discharge tests were performed at a current density of 0.5 A·g^−1^ with a voltage limit of 1.5 V. A total of 3000 charge–discharge cycles were carried out at −10 °C. Based on the obtained results, the relative change in the discharge capacitance was calculated by normalizing the capacitance values to that obtained during the first discharge cycle.

#### 4.4.6. Gel Fraction Analysis

The gel content of the crosslinked polymer network was determined using Soxhlet extraction. The extraction was performed in ethanol under reflux conditions for 24 h to ensure the removal of soluble fractions from the polymer matrix. After extraction, the remaining insoluble fraction was carefully collected and subsequently dried at 80 °C under reduced pressure until constant mass was achieved. The gel fraction was calculated as the percentage ratio of the dry mass of the insoluble residue to the initial sample mass, taking into account the content of the electrolyte in the formulation.

## Figures and Tables

**Table 1:** Compositions of the photocurable mixtures used in the work (where f*_TtEGDA_*—denotes the fraction of carbon–carbon double bonds contributed by the crosslinking monomer relative to the total number of polymerizable double bonds in the formulation).

Sample Name	Electrolyte *, wt%	TtEGDA, wt%	AAm, wt%	f_TtEGDA_	HCPK, wt%
HPE_70	69.9	0.8	29.1	0.0127	0.2
HPE_80	79.8	1.4	18.6	0.0342	0.2
HPE_85	84.8	1.5	13.5	0.0603	0.2
HPE_90	89.8	2.0	8.0	0.1052	0.2

* 2.5 mol [Chol][LA] per kg H_2_O.

**Table 2:** Ionic conductivity (σ) at 25 °C and VTF equation parameters of the studied HPEs with different molar ratios of electrolyte (σ_∞_—pre-exponential parameter in mS·cm^−1^, E_a,VTF_—pseudo-activation energy in kJ·mol^−1^). Fitting method—maximize linearity. For all regression curve determination coefficients R^2^ > 0.999.

Sample	σ, mS·cm^−1^ at 25 °C	T_0_, K	σ_∞_, mS·cm^−1^	B, K	E_a,VTF_, kJ·mol^−1^
HPE_70	10.5	164	736	−567	4.71
HPE_80	12.9	169	556	−487	4.05
HPE_85	18.2	168	687	−472	3.92
HPE_90	22.3	166	704	−459	3.82
electrolyte *	36.6	164	1058	−451	3.74

* 2.5 mol [Chol][LA] per kg H_2_O.

## Data Availability

Not applicable.
